# Dietary docosahexaenoic acid (DHA) downregulates liver DHA synthesis by inhibiting eicosapentaenoic acid elongation

**DOI:** 10.1016/j.jlr.2024.100548

**Published:** 2024-04-20

**Authors:** Adam H. Metherel, Rodrigo Valenzuela, Brinley J. Klievik, Giulia Cisbani, Ruxandra D. Rotarescu, Melissa Gonzalez-Soto, Céline Cruciani-Guglielmacci, Sophie Layé, Christophe Magnan, David M. Mutch, Richard P. Bazinet

**Affiliations:** 1 Department of Nutritional Sciences, University of Toronto, Toronto, ON, Canada; 2 Department of Nutrition, University of Chile, Santiago, Chile; 3 Department of Human Health and Nutritional Sciences, University of Guelph, Guelph, ON, Canada; 4 BFA, UMR8251, CNRS, Université Paris Cité, Paris, France; 5 INRA, Bordeaux INP, NutriNeuro, Université de Bordeaux, Bordeaux, France

**Keywords:** dietary fat, kinetics, liver, nutrition, omega-3 fatty acids, enzyme inhibition, polyunsaturated fatty acid, fatty acid metabolism, elongation of very long-chain

## Abstract

DHA is abundant in the brain where it regulates cell survival, neurogenesis, and neuroinflammation. DHA can be obtained from the diet or synthesized from alpha-linolenic acid (ALA; 18:3n-3) via a series of desaturation and elongation reactions occurring in the liver. Tracer studies suggest that dietary DHA can downregulate its own synthesis, but the mechanism remains undetermined and is the primary objective of this manuscript. First, we show by tracing ^13^C content (δ^13^C) of DHA via compound-specific isotope analysis, that following low dietary DHA, the brain receives DHA synthesized from ALA. We then show that dietary DHA increases mouse liver and serum EPA, which is dependant on ALA. Furthermore, by compound-specific isotope analysis we demonstrate that the source of increased EPA is slowed EPA metabolism, not increased DHA retroconversion as previously assumed. DHA feeding alone or with ALA lowered liver elongation of very long chain (ELOVL2, EPA elongation) enzyme activity despite no change in protein content. To further evaluate the role of ELOVL2, a liver-specific *Elovl2* KO was generated showing that DHA feeding in the presence or absence of a functional liver ELOVL2 yields similar results. An enzyme competition assay for EPA elongation suggests both uncompetitive and noncompetitive inhibition by DHA depending on DHA levels. To translate our findings, we show that DHA supplementation in men and women increases EPA levels in a manner dependent on a SNP (rs953413) in the *ELOVL2* gene. In conclusion, we identify a novel feedback inhibition pathway where dietary DHA downregulates its liver synthesis by inhibiting EPA elongation.

The brain is enriched with the omega-3 (n-3) PUFA, DHA, where it either directly or upon conversion to a series of bioactive metabolites, regulates several important aspects of brain function (1, 2). DHA is a precursor to both n-docosahexaenoylethanolamine (synaptamide) and oxylipins, including the specialized proresolving lipid mediators which have been shown to affect neurodevelopment (3, 4), inflammation (5, 6, 7), and cell survival (8, 9). The brain can either obtain DHA from the diet directly or after desaturation and elongation of its 18-carbon essential PUFA precursor, alpha-linolenic acid (ALA, 18:3n-3). While many tissues have the capacity to synthesize DHA, their ability to do so is generally considered low, especially within the brain, and they rely on synthesis of DHA from the liver (10, 11, 12, 13). Interestingly, the liver, but not the brain, can upregulate DHA synthesis in response to low dietary DHA, albeit the mechanism is not known (14, 15). Furthermore, since 1985 (16), DHA supplementation has been widely observed to increase EPA levels in humans (16, 17, 18, 19, 20, 21, 22, 23, 24) and rodents (25, 26, 27, 28, 29, 30, 31, 32), and this response was believed to be the result of an increased flux though the retroconversion pathway discovered in 1969 (33) that involves partial β-oxidation of DHA in the peroxisomes to form EPA.

Compound-specific isotope analysis (CSIA) has emerged as a valuable tool for the investigation of nutrition and metabolism and has recently been reviewed (34). Briefly, ^13^C abundance (δ^13^C) in plants varies naturally depending on the photosynthetic pathways present, with C4 photosynthetic plants (i.e., sugar cane and corn) having higher δ^13^C values than C3 photosynthetic plants (i.e., flax and canola), with the latter making up the vast majority of all plant species (35). These natural variations in δ^13^C of nutrients—including fatty acids—of plants and the tissues of animals consuming them are what enables CSIA-driven nutrition and metabolic studies via high precision GC-combustion-isotope ratio mass spectrometry (C-IRMS). Using CSIA, we recently demonstrated that the previously accepted mechanisms explaining the increase of EPA with DHA supplementation was inaccurate (24). Specifically, young adults were supplemented with 3 g/day of DHA containing high δ^13^C-DHA levels (−23.6 ± 0.2, milliUrey (mUr) ± SEM) relative to low baseline plasma δ^13^C-EPA levels in the participants. As expected, DHA supplementation increased plasma EPA concentrations by 130% over 12 weeks; however, δ^13^C-EPA levels did not change from baseline (−31.0 ± 0.3) to post supplementation (−30.8 ± 0.2) suggesting that ALA, with a baseline plasma δ^13^C-ALA of −30.8 ± 0.3, was contributing to the rise in DHA. This translational work supported a previous study in which Long-Evans rats fed either an ALA + DHA diet or an ALA only diet for 8 weeks demonstrated no differences in liver δ^13^C-EPA values (−26.3 ± 0.4 and −25.9 ± 0.4, respectively) despite the significantly higher δ^13^C values of DHA in the diet of −22.3 ± 0.3 (36).

Collectively, this novel finding that ALA is a metabolic source for the higher EPA in human plasma and rat liver following DHA intake suggests a slowing of EPA metabolism. In this study, we aim to identify if the mechanism for slowed EPA metabolism serves to regulate brain DHA via inhibiting DHA synthesis in the liver. In the present study, we (1) find that in response to low DHA feeding, the brain receives DHA synthesized from ALA, (2) feed ALA, DHA, or ALA + DHA to WT (BALB/c), as well as control, and liver-specific elongation of very long chain 2 (*Elovl2*) KO mice (C57Bl/6J), to demonstrate that DHA combined with ALA is required to increase EPA in a manner indicative of ELOVL2 inhibition, (3) use an enzyme competition assay to demonstrate the uncompetitive and noncompetitive inhibition by DHA on elongation of EPA, and (4) perform a secondary analysis on human plasma from DHA supplemented men and women to identify a SNP in *ELOVL2* that results in a larger increase in EPA levels following DHA supplementation. Collectively this work identifies that EPA elongation in the liver is downregulated by dietary DHA to inhibit DHA synthesis.

## Materials and methods

### Ethics statement

All animal protocols and procedures were performed in alignment with the policies of the Canadian Council on Animal Care and were approved by the University of Toronto Animal Ethics Committee. The human randomized control trial was approved by the Human Research Ethics Board at the University of Guelph, abides by the Declaration of Helsinki principles and was registered at clinicaltrials.gov (NCT03378232).

### Study design

#### Mouse feeding study

Twenty-four 28-day-old male BALB/c mice were ordered from Charles River Laboratories (St. Constance, QC) and placed on 2% ALA in total fat diet (ALA) for 4 weeks. After this initial 4 weeks of ALA feeding, mice were placed on one of the three diets for an additional 4 weeks: (1) maintained on the 2% ALA diet, (2) switched to a 2% DHA in total fat (DHA) diet, or (3) switched to a 2% ALA + 2% DHA in total fat (ALA + DHA) diet. All diets were modified from the AIN-93G custom low n-3 PUFA diets (Dyets, Inc, Bethlehem, PA) used previously (31, 36, 37, 38). The diets contained 10% lipids by weight consisting of 32.8% safflower oil, 63.2% hydrogenated coconut oil, and 4% added oils (a combination of ALA, DHA, and oleate ethyl esters) purchased from Nu-Chek Prep, Inc (Elysian, MN). The 2% individual ALA and 2% DHA diets also contained 2% oleate ethyl ester to balance the additional n-3 PUFA present in the ALA + DHA diet.

Diet fatty acid compositions were determined by GC-flame ionization detection (FID) to contain 2.20 ± 0.02% ALA (ALA-only diet), 1.85 ± 0.01% DHA in the DHA-only diet, and 2.15 ± 0.01 ALA and 1.92 ± 0.02% DHA in the ALA + DHA diet. Furthermore, δ^13^C values were determined by GC-C-IRMS to be −31.2 ± 0.4 mUr for ALA, −14.0 ± 1.3 mUr for DHA, and −34.0 ± 0.8 mUr for ALA and −11.0 ± 0.3 mUr for DHA in the ALA, DHA and ALA + DHA diets, respectively (see supplemental Table S1 for complete fatty acid composition). At 12 weeks of age and following the 8-week feeding period, mice were anesthetized under isoflurane, blood was collected by cardiac puncture, and animals were perfused with cold (4°C) phosphate-buffered saline into the left ventricle. Serum and livers were collected and immediately frozen on dry ice before storage at −80°C prior to determining fatty acid levels, gene expression, protein content and enzyme activity.

#### Liver-specific Elovl2 KO mouse feeding study

Six C57Bl/6J mice (two males and four females) were received from the Université Paris Cité. All mice were floxed (*lox/lox*) for the *Elovl2* gene, with male mice expressing *Cre* (+/−) in the liver under albumin promotor, and female mice not expressing *Cre* (−/−). Liver *Elovl2* KO specificity was confirmed by gene expression prior to shipment in comparison to kidney *Elovl2* expression. Upon arrival at the University of Toronto, mice were quarantined for approximately 2 weeks and then harem bred (one male and two females per harem). At weaning (21 days), pups were genotyped for *Cre* and at 28-days old pups were placed onto the same 8-week dietary feeding protocol described for the BALB/c mouse feeding study. After 8 weeks of feeding, male mice were perfused, and serum, brains, and livers were collected and stored as described above for the BALB/c mice. Mice from the same litters were placed in different dietary groups by genotype with *lox/lox* mice negative for *Cre* (−/−) included as the control group.

#### Enzyme competition study

Eight-week-old male BALB/c mice were ordered from Charles River Laboratories and placed on a standard Teklad 2918 chow diet (Envigo, Indianapolis, IN) for 6 weeks. As reported previously, fatty acid composition of the diet, as % weight in total fatty acids was 6.2% ALA, 11.8% 16:0, 3.2% 18:0, 1.1% 18:1n-7, 20.0% 18:1n-9, and 53.5% 18:2n-6 (39). Following 6 weeks, 12-week-old mice were perfused with cold-saline and livers collected as described above for future analysis of liver enzyme activity for the conversion of EPA to n-3 docosapentaenoic acid (DPAn-3, 22:5n-3) (ELOVL2/5).

#### Human DHA supplementation study

Human plasma samples from males and females (n = 15 and 14, respectively) of a previously published randomized control trial (24, 40) were used to assess the role of the rs953413 (*ELOVL2*) and rs174537 fatty acid desaturase 1 (*FADS1*) SNP on changes in EPA levels following 12 weeks supplementation with 3 g/day of fish oil derived DHA triglycerides (72.3% purity). Small amounts of n-3 PUFA impurities were present in the form of 0.2% ALA, 1% EPA, and 1.5% DPAn-3, while all other impurities were saturates (4%), monounsaturates (16%) or linoleic acid (2%) (24). Methodological and participant information has been reported in detail previously (24, 40).

### Fatty acid analyses

#### Lipid extraction and preparation of fatty acid methyl esters

Lipids were extracted from 300 μl of human plasma, 100 μl of mouse serum, and 50 mg of mouse brain and liver in 2:1 chloroform:methanol by a modified Folch method (41). Docosatrienoic acid (22:3n-3) ethyl ester (Nu-Chek Prep) was included at 10, 5, and 25 μg, respectively, as internal standard for fatty acid quantification. After homogenization, lipid and aqueous phases were separated with the addition of 0.88% KCl, centrifuged at 500 *g* for 5 min, and the bottom lipid-containing chloroform phase was isolated. The resulting total lipid extracts for plasma/serum and an aliquot of the liver total lipid extracts were dried under a stream of nitrogen and transesterified with 14% boron trifluoride in methanol for 1 h at 100 °C. The samples were cooled to room temperature, 1 ml of water and hexane were added, vortexed, and centrifuged at 500 *g* for 5 min. The top hexane layer containing fatty acid methyl esters (FAMEs) were isolated, dried under nitrogen, resuspended in 100–200 μl of heptane, analyzed by GC-FID, recapped, and subsequently analyzed by GC-C-IRMS.

#### Gas chromatography

FAMEs generated from human plasma were analyzed on a Varian 430 GC-FID (SCION Instruments, Goes, NL) equipped with a SP-2560 100 m × 0.25 mm i.d. × 0.20 μm film thickness capillary column (MilliporeSigma, Burlington, MA), as previously described (24, 37). FAMEs generated from mouse serum, brain, and liver were analyzed on the same GC-FID, equipped instead with a DB-FFAP 30 m × 0.25 mm i.d. × 0.25 μm film thickness capillary column (J&W Scientific from Agilent Technologies, Mississauga, ON). FAMEs (1 μl) were introduced into the GC-FID by a Varian CP-8400 autosampler into the injector heated at 250°C with a split ratio of 30:1. Initial oven temperature was 50°C with a 1.0 min hold followed by a 30°C/min ramp to 130°C, a 10°C/min ramp to 175°C, a 5.0°C/min ramp to 230°C with a 9.5 min hold, and then a 50°C/min ramp up to 240°C with an 11.1 min hold at the end. The FID temperature was 300°C with air and helium make-up gas flow rates of 300 and 25 ml/min, respectively, and a sampling frequency of 40 Hz. Peaks were identified by retention times through comparison to an external mixed standard sample (GLC-462, Nu-Chek Prep).

FAMEs generated from mouse serum, brain, and liver were analyzed for carbon-13 content (δ^13^C) on a Thermo MAT253 C-IRMS (Thermo Fisher Scientific, Bremen, Germany), injected by a TriPlus RSH autosampler onto a SP-2560 capillary column affixed to a Trace 1310 GC. Fatty acids enter the combustion reactor and are quantitatively combusted to CO_2_, which enters the MAT253 IRMS via a ConFlo IV continuous flow interface providing the δ^13^C of the CO_2_, and therefore the FAME of interest. All additional methodological details relevant to δ^13^C analysis, such as analysis, reporting of δ^13^C values, isotopic normalization, and methyl group corrections are as previously described in detail (24, 36).

### Gene and enzyme analyses

#### Liver-specific Elovl2 KO mouse genotyping

DNA from the ear punches collected from weaned mice at 21 days were extracted with REDExtract-N-Amp Tissue PCR Kit (MilliporeSigma). Briefly, 100 μl of extraction solution and 25 μl of tissue preparation solution were added to ear punches, vortexed, incubated at room temperature for 10 min, and at 95°C for 3 min. Samples were removed from heat, 100 μl of neutralization solution immediately added and vortexed. DNA extracts were then stored at −20°C until further analysis. DNA was amplified using the FastStart Taq DNA Polymerase kit (Roche Diagnostics, Mississauga, ON). Briefly, each sample/reagent mixture for amplification included 10.8 μl water, 2 μl PCR reaction buffer 10× with 20 mM MgCl_2_, 0.4 μl dNTP nucleotide mix, 0.16 μl Taq DNA polymerase, 1.3 μl each of *Cre* (F-5′ GATGGCAAACATACGCAAGG, R-5′ CCCTGAACATGTCCATCAGG) and β-*actin* (F-5′ GCCTCATGCCATTCTACGAC, R-5′ CGAATACTTGCGTTCTGGGG) forward and reverse primers, and 1.5 μl of extracted DNA material. Using the Biometra Professional Gradient System (Montreal Biotech Inc, Kirkland, QC), the PCR mixtures were then incubated at 95°C for 4 min, followed by 40 cycles of 95°C for 0.5 min, 58°C for 0.5 min, and 72°C for 1.0 min, and a final hold at 72°C for 7 min to amplify the *Cre* and β-*actin* target and positive control genes, respectively. To each mixture, 5 μl of Safe-Green fluorescent dye (Abcam, Cambridge, UK) was added, mixed, and 10 μl was added to 1.5% agarose gel wells and run by gel electrophoresis for 45 min at 100 V. Agarose gels were visualized under UV light for *Cre* (386 bp) and β-*actin* (502 bp) bands in relation to 100–1,500 bp reference DNA ladder (FroggaBio, Concord, ON) (supplemental Fig. S1).

#### Human genotyping

DNA was extracted from whole blood using the Qiagen PAXgene Blood DNA kit (Qiagen, Toronto, ON) and genotyping performed using the Sequenom MassArray platform, as previously described (42, 43). The genotypes for SNP rs953413 (*ELOVL2*) and rs174537 (*FADS1*) were identified for all participants. Deviations from Hardy-Weinberg equilibrium were tested for each SNP for all participants and within male and female groups using a Chi-square test. As previously reported (42), five samples were selected randomly for replication with 100% accordance achieved.

#### Liver gene expression and protein masses of BALB/c mice

Total RNA was extracted using TRIzol Reagent (Invitrogen, Massachusetts, USA) for tissue homogenization and the RNeasy Mini Kit (Qiagen, Mississauga, ON, CA), as per manufacturer's instructions. RNA concentration and purity were determined using a NanoDrop™ 2000 Spectrophotometer (Thermo Fisher Scientific, Wilmington, USA). Only samples with sufficient RNA concentration and purity ratios (260/230 and 260/280) between 1.8 and 2.2 were used for gene expression analyses. One microgram of total RNA was used to synthesize cDNA using the Applied Biosystems™ High-Capacity cDNA Reverse Transcription Kit (Thermo Fisher Scientific), as per manufacturer's instructions. Reverse transcription-quantitative PCR (RT-qPCR) was conducted on a Bio-Rad CFX96 Real-Time system. The primers used for RT-qPCR were designed online using the Roche Universal Probe Library and Assay Design Center. A total reaction volume per sample (10 μl) was made, consisting of 5 μl SsoFast Evagreen Supermix (Bio-Rad Laboratories), 2.5 μl cDNA template, 0.2 μl of 10 μM forward and reverse primer mixture, and 2.3 μl nuclease-free water. The following cycling conditions were used: one denaturing cycle at 95°C for 30 s, followed by 40 cycles of 95°C for 4 s and 55.9°C for 4 s. All reactions were run in triplicate. Subsequently, 18S was used as a housekeeping gene for normalization. Results were quantified using the ΔΔCt method.

Protein masses (ng/g) of FADS2, FADS1, ELOVL2, and ELOVL5 enzymes were determined by commercially available sandwich enzyme-linked immunosorbent assay kits (MyBioSource, Inc, San Diego, CA, USA) according to the manufacturer's instructions. Briefly, 100 μl of diluted liver homogenate was added to sample wells, the plates were sealed and then incubated at 37°C for 90 min. The plates were then washed, biotin-labeled antibody added to the wells, incubated at 37°C for 60 min and then washed. HRP-streptavidin conjugate was then added, incubated, and washed. 3,3′,5,5′-tetramethylbenzidine substrate was then added, incubated at 37°C for 10–20 min, the reaction stopped, and absorbance was read at 450 nm and compared to a standard curve.

#### Liver enzyme activities of BALB/c mice

Perfused livers from the BALB/c mouse feeding study frozen on dry ice (∼500 mg) were homogenized in a buffer solution pH 7.9 containing 10 mmol/l Hepes, 1.0 mmol/l EDTA, 0.6% Nonidet P-40, 150 mmol/l NaCl, and protease inhibitors (1 mmol/l phenylmethylsulfonyl fluoride, 1 μg/ml aprotinin, 1 μg/ml leupeptin, and 1 mmol/l orthovanadate). Liver homogenates were centrifuged at 4 °C, first at 2,000 *g* for 30 s, followed by centrifugation of the supernatants at 5,000 *g* for 5 min, and finally at 100,000 *g* for 60 min, to obtain the microsomal fractions for the assessment of desaturase and elongase activities.

Desaturase and elongase activities were assayed using 1 ml of incubation medium containing 4 μmol ATP, 0.1 μmol coenzyme-A, 1.28 μmol NADPH, 2.42 μmol N-acetylcysteine, 0.5 μmol nicotinamide, 5 μmol MgCl_2_, 62.5 μmol NaF, and 62.5 μmol phosphate buffer pH 7, added with 100 nmol albumin-bound n-3 PUFA precursor and 1 mg protein of cytosolic extract to a final total reaction mixture volume of 2 ml incubated at 37°C for 30 min with shaking. Each reaction represents the equivalent of between 60 and 80 mg of liver tissue. When determining elongase activity, 5 mmol of malonyl-CoA was included in the reaction mixture. FADS2 enzyme activity was determined by the amount of 18:3n-3 (ALA) converted to 18:4n-3 (SDA), FADS1 activity by the amount of 20:4n-3 (ETA) converted to 20:5n-3, ELOVL2 activity by the amount of 22:5n-3 (DPAn-3) converted to 24:5n-3 (n-3 tetracosapentaenoic acid), ELOVL5 activity by the amount of 18:4n-3 converted to 20:4n-3 (ETA), and ELOVL2/5 activity by the amount of 20:5n-3 converted to 22:5n-3 (44).

Each specific reaction was stopped by adding 6 ml of a chloroform:methanol mixture (2:1 v/v). Heptadecanoic acid (17:0; purity ≥99%) was added (20 μg) as internal standard. To determine the levels of products or precursors achieved after incubation, lipids were extracted and derivatized to FAME and levels determined by GC-FID, as described earlier. From GC-FID results, desaturase and elongase enzyme activities were measured as net increase of n-3 PUFA product produced from precursor n-3 PUFA, calculated from the differences between baseline values prior to incubation and those obtained after 30 min incubation. Results are expressed as ng/min/mg protein (45).

#### Enzyme competition assay by DHA for the ELOVL2/5 conversion of EPA to DPAn-3

To determine the effect of DHA on the ELOVL2/5 reaction activity (20:5n-3 conversion to 22:5n-3) we performed the same microsomal isolation and enzyme activity reaction described previously in mice for the enzyme competition study. The enzyme competition assays were prepared using DHA as the inhibitor at different concentrations (0, 5, 10, 25, 50, and 100 μmol) in the presence of EPA, the elongase reaction precursor, at different concentrations (0, 50, 100, 150, and 200 μmol). The ELOVL2/5 product (22:5n-3) concentrations as a result of the reaction, were determined as ng/min/mg protein and used to generate Michaelis-Menten curves. To evaluate the effect of DHA in comparison to a control fatty acid, palmitic acid (16:0) and DHA were compared as inhibitors of ELOVL2/5 at increasing concentrations of both fatty acids (0, 25, 50, and 100 μmol) in the presence of 100 μmol EPA.

#### Statistical analyses

ANOVAs and accompanying Tukey’s post hoc tests were performed with IBM Statistics 24 software (IBM, Armonk, NY, https://www.ibm.com/products/spss-statistics). Data that were not normally distributed as determined by the Shapiro-Wilk test were log transformed prior to ANOVA analysis. Michaelis-Menten curves for the enzyme competition assay were developed using GraphPad Prism 9.1 (GraphPad Software, San Diego, CA, https://www.graphpad.com/). Significance for all statistical analysis was determined at *P* < 0.05. All data are presented as means ± SEM.

## Results

### Brain DHA concentrations and δ^13^C levels from control mice fed ALA, DHA, or ALA + DHA diets

**Fig. 1:**
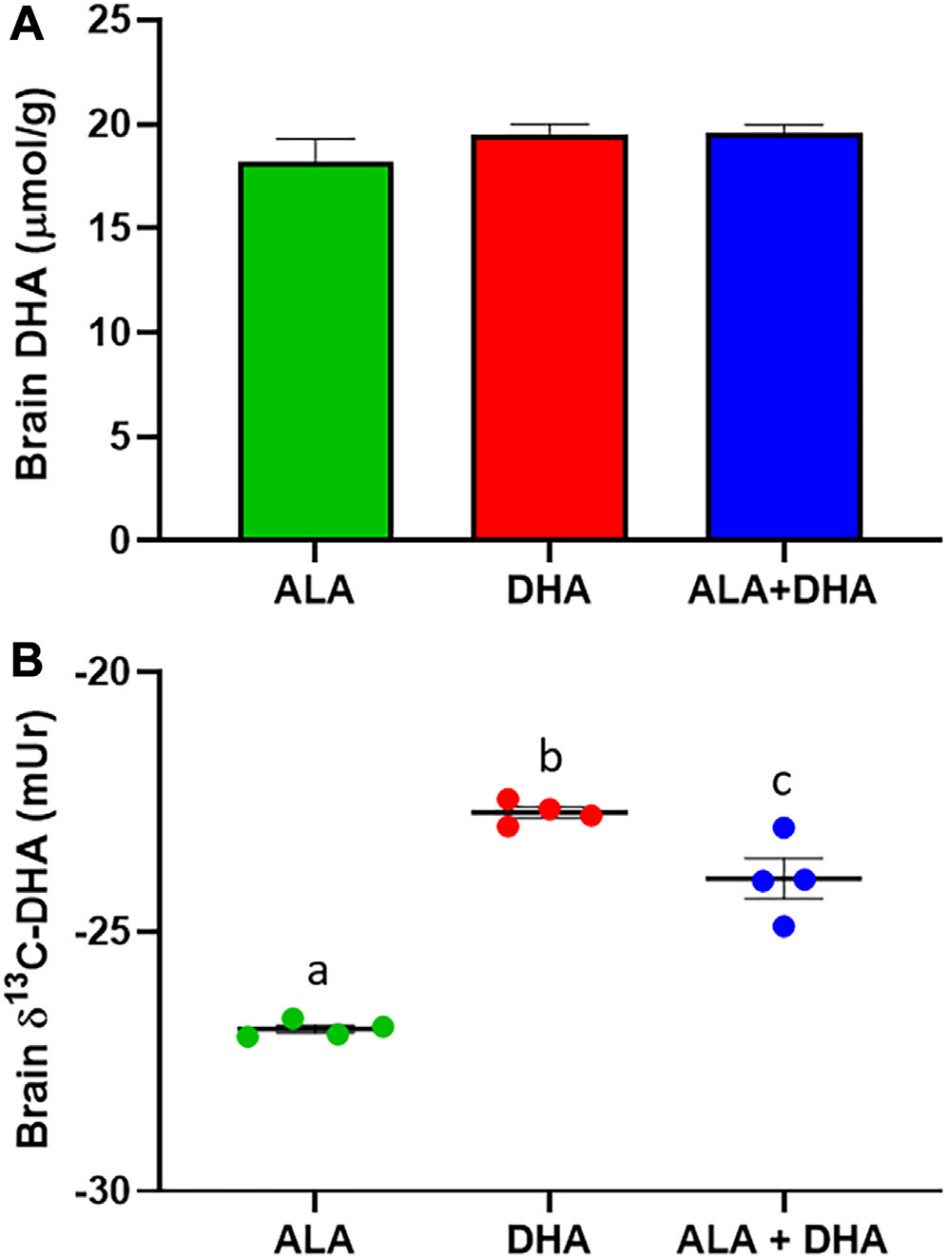
Brain DHA (A) concentrations and (B) carbon-13 levels (δ^13^C) of control mice fed an ALA only, DHA only and ALA + DHA diet. Normality was determined by Shapiro-Wilk test for normality and nonnormally distributed data were log transformed prior to further statistical analysis. Different letters (a, b, and c) represent statistically significant differences (*P* < 0.05) between diets and were determined by one-way ANOVA followed by a Tukey’s HSD post hoc test. All values are reported in means ± SEM (n = 8). δ^13^C, carbon-13 abundance; ; ALA, α-linolenic acid, 18:3n-3; DHA, docosahexaenoic acid, 22:6n-3; HSD, honestly significant difference.

In order to test how the brain obtains DHA when DHA is absent or present in the diet, the control *Elovl2* KO mice underwent 4 weeks of feeding an ALA only diet followed by an additional 4 weeks of feeding the ALA only, DHA only, and ALA + DHA diets. Tukey’s post hoc analysis did not result in any significant differences (*P* > 0.05) in the brain DHA concentrations (18.2 ± 0.6, 19.5 ± 0.2 and 19.6 ± 0.2, μmol/g ± SEM, respectively) following a significant one-way ANOVA (*P* < 0.05) (Fig. 1A). However, brain δ^13^C-DHA levels were significantly different (*P* < 0.01) between all groups with DHA only (−22.7 ± 0.1, mUr ± SEM) > ALA + DHA (−24.0 ± 0.4) > ALA only diets (−26.9 ± 0.1) (Fig. 1B).

### Serum and liver n-3 PUFA concentrations of BALB/c mice fed ALA, DHA, or ALA + DHA

**Fig. 2:**
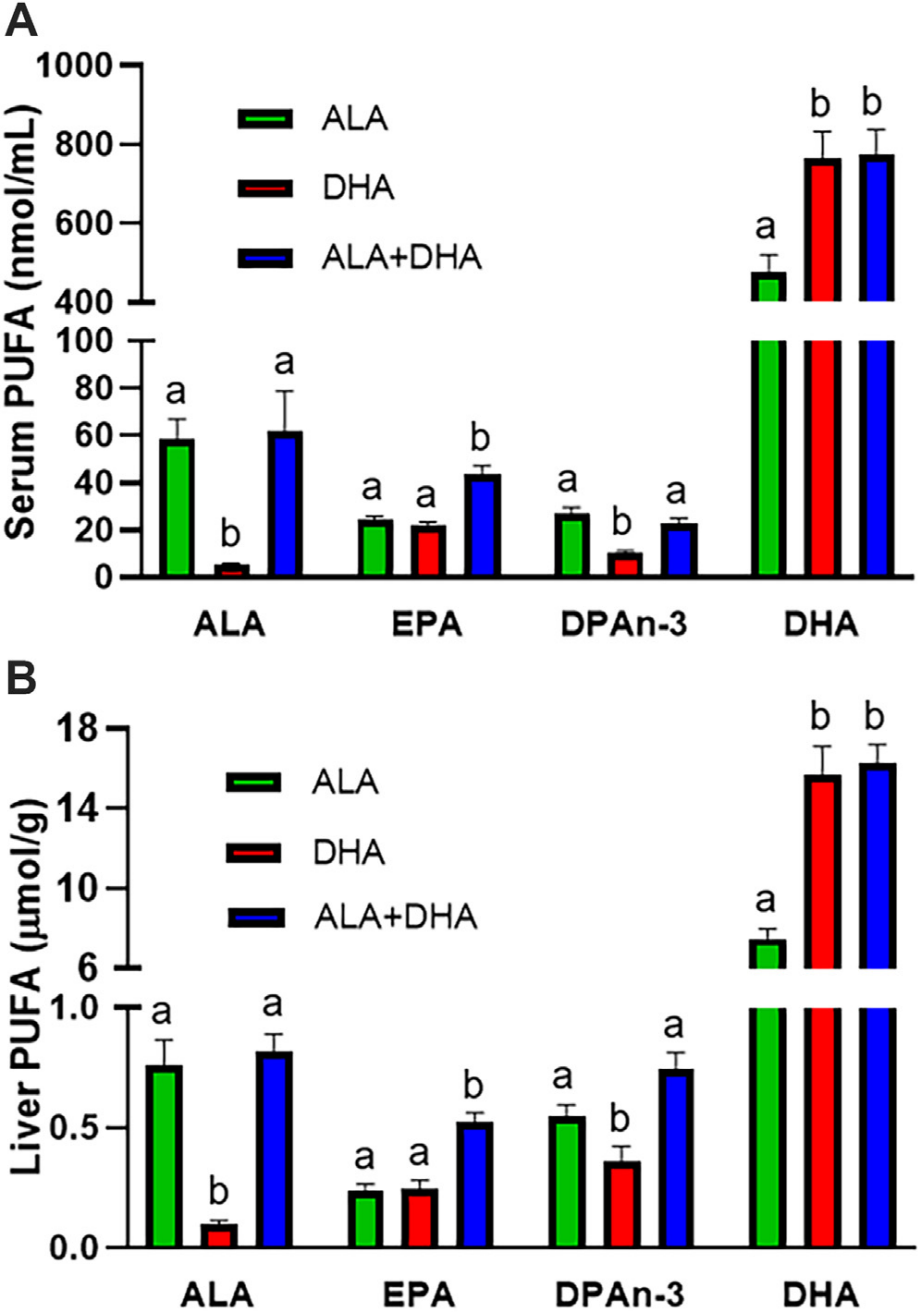
N-3 PUFA concentrations in male BALB/c mice fed 4 weeks of: (1) ALA only (ALA), (2) DHA only (DHA) or (3) ALA + DHA diets following a 4-week ALA run-in diet. A: Serum and (B) liver. Normality was determined by Shapiro-Wilk test for normality and nonnormally distributed data was log transformed prior to further statistical analysis. Different letters (a, b, and c) represent statistically significant differences (*P* < 0.05) between diets for a specific fatty acid and were determined by one-way ANOVA followed by a Tukey’s HSD post hoc test. All values are reported in means ± SEM (n = 8). ALA, α-linolenic acid, 18:3n-3; DHA, docosahexaenoic acid, 22:6n-3; DPAn-3, n-3 docosapentaenoic acid, 22:5n-3; EPA, eicosapentaenoic acid, 20:5n-3; HSD, honestly significant difference.

**Fig. 3:**
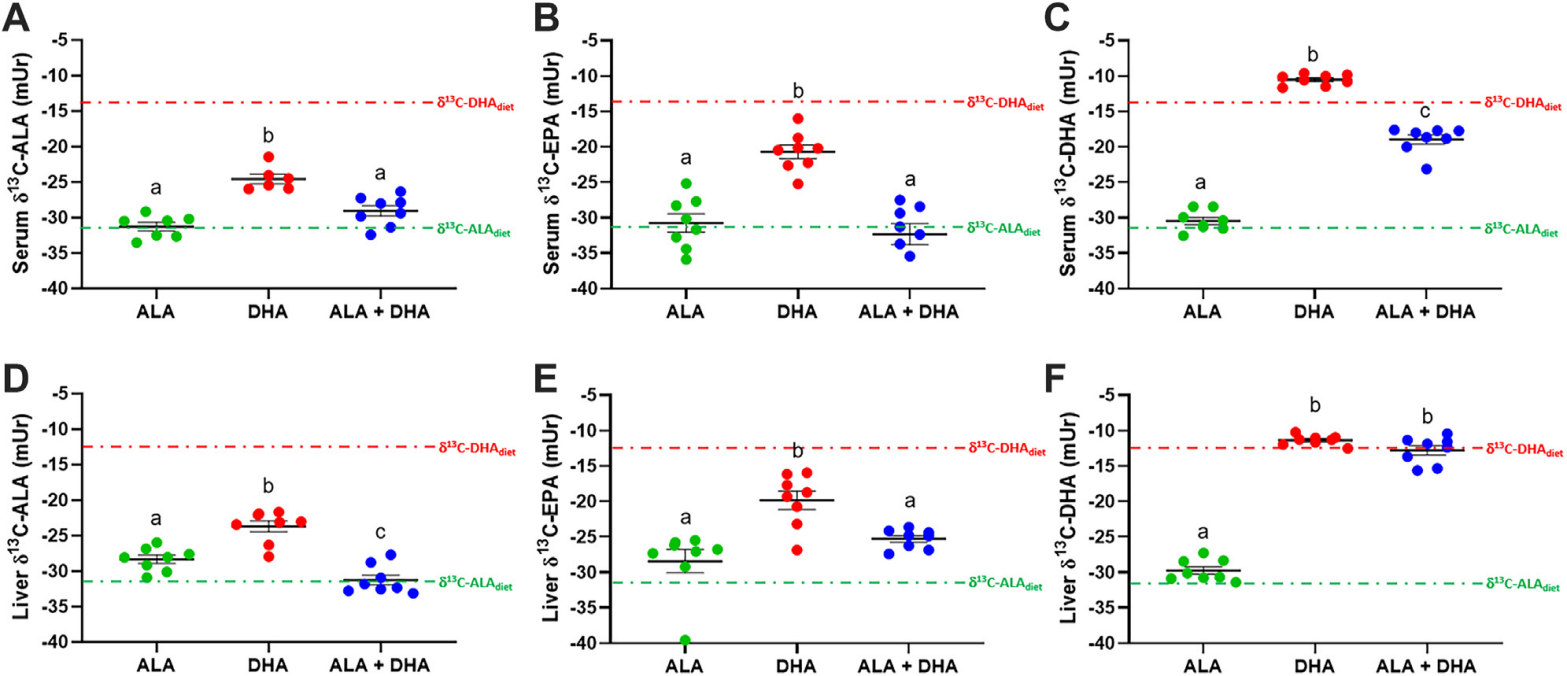
ALA, EPA, and DHA carbon-13 levels (δ^13^C) of male BALB/c mice fed 4 weeks of (1) ALA only (ALA), (2) DHA only (DHA) or (3) ALA + DHA diets following a 4-week ALA run-in diet. A: serum ALA, (B) serum EPA, (C) serum DHA, (D) liver ALA, (E) liver EPA, and (F) liver DHA. Normality was determined by Shapiro-Wilk test for normality and nonnormally distributed data were log transformed prior to further statistical analysis. Different letters (a, b, and c) represent statistically significant differences (*P* < 0.05) between diets and were determined by one-way ANOVA followed by a Tukey’s HSD post hoc test. All values are reported in means ± SEM (n = 6–8). δ^13^C, carbon-13 abundance; δ^13^C-ALA_diet_, mean δ^13^C (mUr) of ALA and ALA+DHA diets; δ^13^C-DHA_diet,_ mean δ^13^C (mUr) of DHA and ALA+DHA diets; ALA, α-linolenic acid, 18:3n-3; DHA, docosahexaenoic acid, 22:6n-3; EPA, eicosapentaenoic acid, 20:5n-3; HSD, honestly significant difference.

Following 4 weeks of feeding an ALA only diet to BALB/c mice and then maintaining animals on the ALA diet or switching to a DHA only or ALA + DHA diet for another 4 weeks, animals were perfused, and serum (Fig. 2A) and liver (Fig. 2B) n-3 PUFA levels determined. In both tissues, ALA levels were 760%–1,160% higher (*P* < 0.05) in mice fed the two ALA-containing diets compared to the DHA only diet that was devoid of ALA. Similarly, DHA levels in both tissues were 59%–117% higher (*P* < 0.05) in the two DHA-containing diets compared to the ALA only diet devoid of DHA. Most importantly, EPA levels were higher (*P* < 0.001) in serum (43.7 ± 3.4, nmol/ml ± SEM) and liver (0.52 ± 0.04, μmol/g ± SEM) of mice fed the ALA + DHA diet for compared to the ones fed ALA only (24.5 ± 1.3 nmol/ml and 0.24 ± 0.02 μmol/g, respectively) and DHA only diet (21.8 ± 1.6 nmol/ml and 0.24 ± 0.04 μmol/g, respectively), suggesting that both ALA and DHA are necessary for the increase in EPA.

### Serum and liver n-3 PUFA carbon-13 content of BALB/c mice fed ALA, DHA, or ALA + DHA

In addition to serum fatty acid concentrations in BALB/c mice (Fig. 2), the δ^13^C of ALA, EPA, and DHA in serum (Fig. 3A–C) and liver (Fig. 3D–F) were determined to identify the sources of each, either synthesis from ALA or from preformed DHA. δ^13^C-ALA levels (mUr ± SEM) in serum were not different (*P* > 0.05) between the ALA (−31.3 ± 0.6 mUr) and ALA + DHA (−29.1 ± 0.7 mUr) fed mice, with both lower (*P* < 0.001) than the DHA only fed mice (−24.6 ± 0.7 mUr). Liver δ^13^C-ALA was different (*P* < 0.05) between all three diet groups where DHA (−23.7 ± 0.8 mUr) > ALA (−28.4 ± 0.6 mUr) > ALA + DHA (−31.3 ± 0.7 mUr). δ^13^C-EPA levels between the ALA and ALA + DHA fed mice in both serum (−30.8 ± 1.3 and −32.3 ± 1.5 mUr, respectively) and liver (−28.4 ± 1.6 and −25.3 ± 0.5 mUr, respectively) were not different (*P* > 0.05). However, δ^13^C-EPA was significantly higher (*P* < 0.01) in the serum (−20.7 ± 1.0 mUr) and liver (−21.8 ± 1.6 mUr) of DHA only group compared to either ALA fed group, revealing that DHA becomes a significant source of EPA only in the absence of ALA. Similar finding were also shown for δ^13^C-DPAn-3 (supplemental Fig. S2). Serum δ^13^C-DHA levels were significantly different (*P* < 0.001) between all diets (DHA > ALA + DHA > ALA; −10.5 ± 0.3, −19.0 ± 0.7 and −30.5 ± 0.5 mUr, respectively), and liver δ^13^C-DHA following DHA (−11.4 ± 0.2 mUr) and ALA + DHA (−12.8 ± 0.7 mUr) feeding were significantly higher (*P* < 0.001) than following ALA feeding (−29.7 ± 0.5 mUr).

### Liver gene expression, protein content, and enzyme activity of BALB/c mice fed ALA, DHA, or ALA + DHA diets

**Fig. 4:**
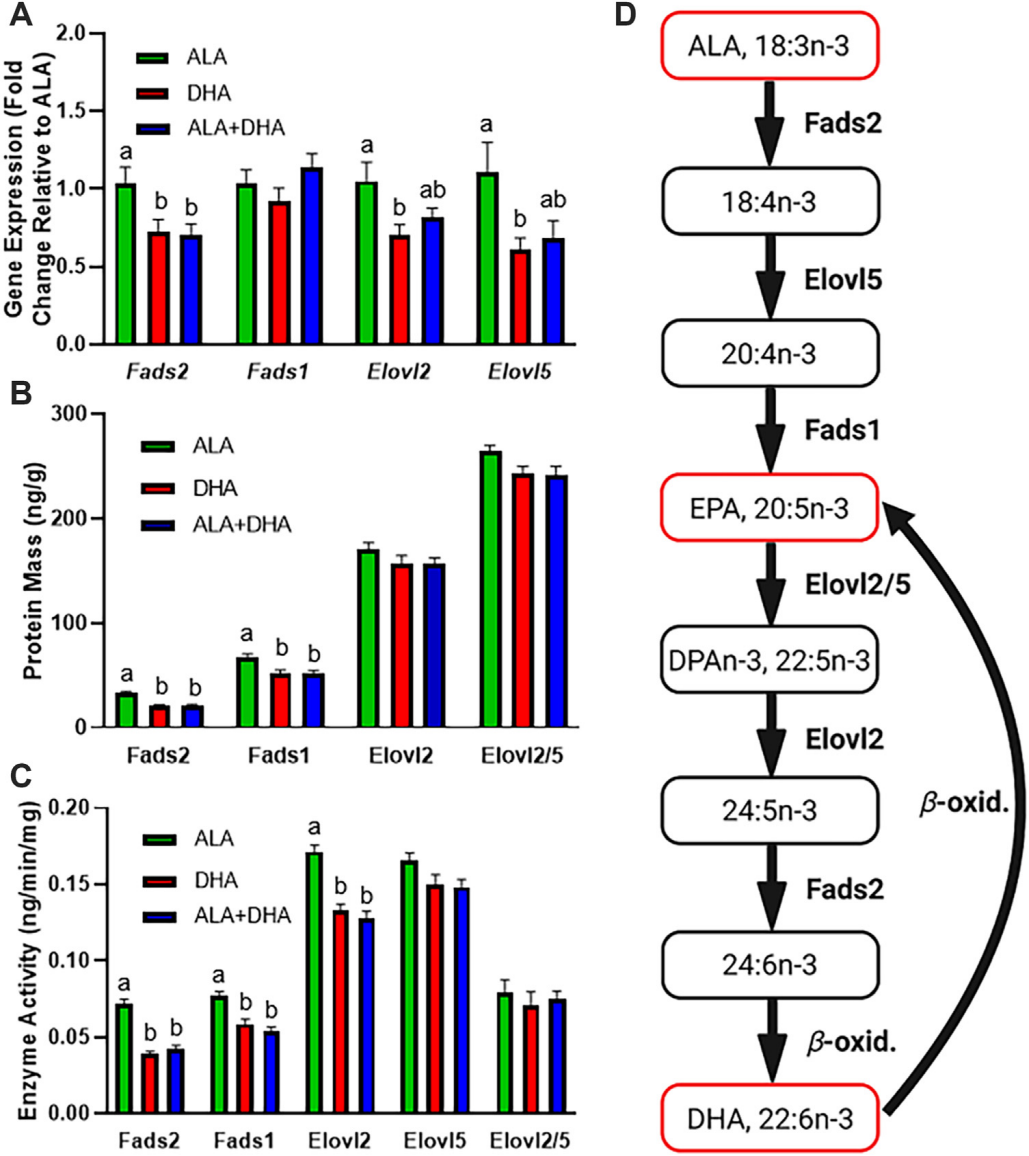
Liver gene expression, protein content, and enzyme activity in male BALB/c mice fed 4 weeks of (1) ALA only (ALA), (2) DHA only (DHA) or (3) ALA + DHA diets following a 4-week ALA run-in diet. A: mRNA expression, (B) protein content, (C) enzyme activity and (D) n-3 PUFA biosynthesis pathway with enzymes. Normality was determined using a Shapiro-Wilk test and nonnormally distributed data were log transformed prior to further statistical analysis. Different letters (a, b, and c) represent statistically significant differences (*P* < 0.05) between diets for a gene, protein or enzyme activity were determined by one-way ANOVA followed by a Tukey’s HSD post hoc test. All values are reported in means ± SEM (n = 6–8). ALA, α-linolenic acid, 18:3n-3; DHA, docosahexaenoic acid, 22:6n-3; Elovl, elongation of very long chain; Fads, fatty acid desaturase; HSD, honestly significant difference.

mRNA expression, protein levels, and enzyme activity (Fig. 4A–C) were measured in the livers of the ALA, DHA, or ALA + DHA fed mice. *Elovl2* mRNA was 33% lower in the DHA group compared to the ALA group (*P* < 0.05), and *Elovl5* was 45% lower in the DHA group compared to ALA group (*P* < 0.05) (Fig. 4A). *Elovl2* and *Elovl5* expression in the ALA + DHA group was not different from ALA or DHA fed mice (*P* > 0.05). Furthermore, *Fads2* mRNA levels were 30% lower in the DHA and 32% lower in the ALA + DHA group compared to the ALA group (*P* < 0.05). There were no differences in mRNA expression for any gene when comparing DHA and ALA + DHA fed mice (*P* > 0.05). No differences were detected in *Fads1* mRNA between the three groups.

DHA only and ALA + DHA feeding resulted in lower (*P* < 0.05) FADS2 (20.8 ± 1.3 and 21.0 ± 1.4, ng/g ± SEM, respectively) and FADS1 (51.0 ± 4.0 and 51.9 ± 3.1 ng/g, respectively) protein content compared to FADS2 (33.0 ± 1.7 ng/g) and FADS1 (67.9 ± 2.4 ng/g) levels in the ALA only fed animals. Protein content of ELOVL2 and ELOVL5 did not differ (*P* > 0.05) between any dietary groups (Fig. 4B).

Liver enzyme activity (ng/min/mg protein ± SEM) was measured in isolated liver microsomes and determined by the rate of conversion of an exogenously supplied precursor to its product (Fig. 4C). Compared to the ALA only fed animals (0.072 ± 0.003 for FADS2, 0.077 ± 0.003 for FADS1 and 0.172 ± 0.004 ng/min/mg for ELOVL2) the enzyme activity was lower for FADS2 (46% and 40%), FADS1 (23% and 30%), and ELOVL2 (22% and 25%) for both the DHA-only and ALA + DHA fed mice, respectively. No differences (*P* > 0.05) in enzyme activity were identified between dietary protocols for ELOVL5 or the ELOVL2/5 reaction (EPA → DPAn-3).

### Serum and liver n-3 PUFA levels of liver-specific *Elovl2* KO mice fed ALA, DHA, or ALA + DHA diets

**Fig. 5:**
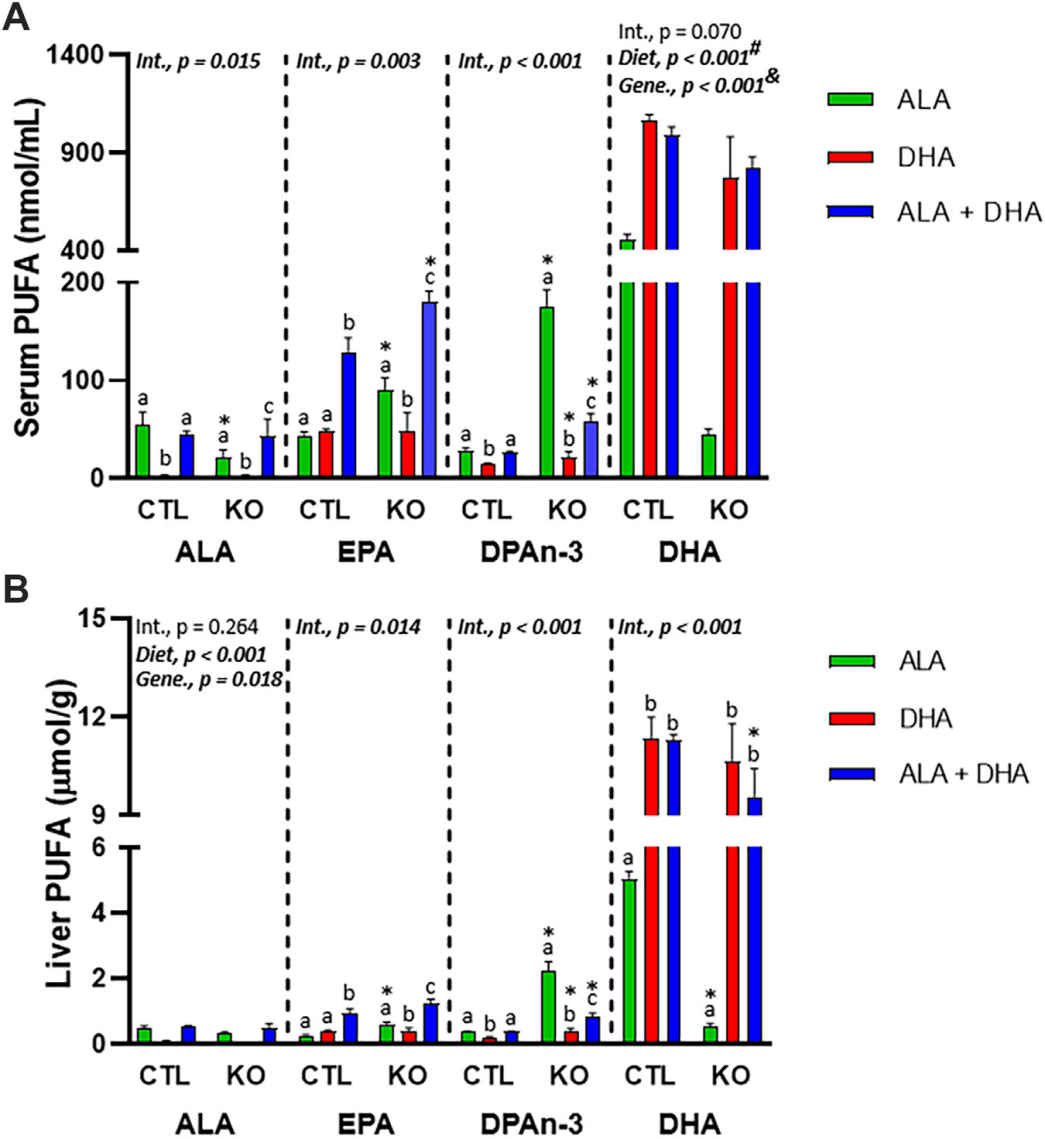
N-3 PUFA concentrations in male liver-specific *Elovl2* KO and control mice fed 4 weeks of: (1) ALA only (ALA), (2) DHA only (DHA) or (3) ALA + DHA diets following a 4-week ALA run-in diet. A: serum and (B) liver. Normality was determined using a Shapiro-Wilk test and nonnormally distributed data were log transformed prior to further statistical analysis. Significant interaction and main effects of genotype and diet were determined by two-way ANOVA. Main effects of diet were further assessed by Tukey’s post hoc test. As a result of a significant interaction effect, different letters (a, b, and c) represent statistically significant differences (*P* < 0.05) between diets for a specific fatty acid within genotype as determined by one-way ANOVA followed by a Tukey’s HSD post hoc test, and (∗) represents statistically significant differences for a specific fatty acid between genotypes and within a diet as determined by independent *t* test. For serum DHA, (^#^) represents a main effect of diet where ALA < (DHA = ALA + DHA) and (^&^) represents a main effect of genotype where CTL > KO. All values are reported in means ± SEM (n = 4–6). ALA, α-linolenic acid, 18:3n-3; DHA, docosahexaenoic acid, 22:6n-3; DPAn-3, n-3 docosapentaenoic acid, 22:5n-3; *Elovl2*, elongation of very long chain 2; EPA, eicosapentaenoic acid, 20:5n-3; HSD, honestly significant difference.

Similar to the feeding study in BALB/c mice, following 4 weeks of feeding an ALA only diet to liver-specific *Elovl2* KO and control mice and then maintaining animals on the ALA diet or switching to a DHA only or ALA + DHA diet for another 4 weeks, animals were euthanized, and serum (Fig. 5A) and liver (Fig. 5B) fatty acid levels determined. A statistically significant interaction effect (genotype × diet) was observed for liver EPA, DPAn-3, and DHA (μmol/g ± SEM) and for serum ALA, EPA, and DPAn-3 (nmol/ml ± SEM). Specifically, liver and serum EPA in the control animals were similar (*P* > 0.05) between the ALA only and DHA only fed animals, and the EPA levels in the ALA + DHA fed animals were higher (*P* < 0.05) than both other diets. Furthermore, liver EPA was higher in the ALA only fed KO mice (0.62 ± 0.02 μmol/g) compared to control (0.26 ± 0.02 μmol/g), with no liver EPA differences between genotypes for the DHA only (0.39 ± 0.04 and 0.38 ± 0.05 μmol/g, respectively) or ALA + DHA fed animals (1.24 ± 0.05 and 0.94 ± 0.13 μmol/g, respectively). In serum, EPA levels were higher in the KO mice of ALA-only (90.6 ± 5.0 nmol/ml) and ALA + DHA fed (181 ± 4.6 nmol/ml) animals compared to control animals (43.8 ± 3.5 and 129 ± 14.4 nmol/ml, respectively). In liver and serum, DPAn-3 levels were 50%–122% higher (*P* < 0.05) in the KO animals of either DHA-fed groups, but 493%–502% higher in the ALA only fed group compared to controls. Finally, liver DHA levels were lower in the KO animals compared to controls in the ALA-only group (0.54 ± 0.04 vs. 5.1 ± 0.2 μmol/g, respectively) and the ALA + DHA group (9.6 ± 0.38 vs. 11.3 ± 0.16 μmol/g, respectively), and serum DHA levels yielded a main effect of genotype where DHA was lower in the KO animals (530 ± 94.4 nmol/ml) compared to controls (836 ± 83.2 nmol/ml).

### Serum and liver n-3 PUFA carbon-13 content of liver-specific *Elovl2* KO mice fed ALA, DHA, or ALA + DHA

**Fig. 6:**
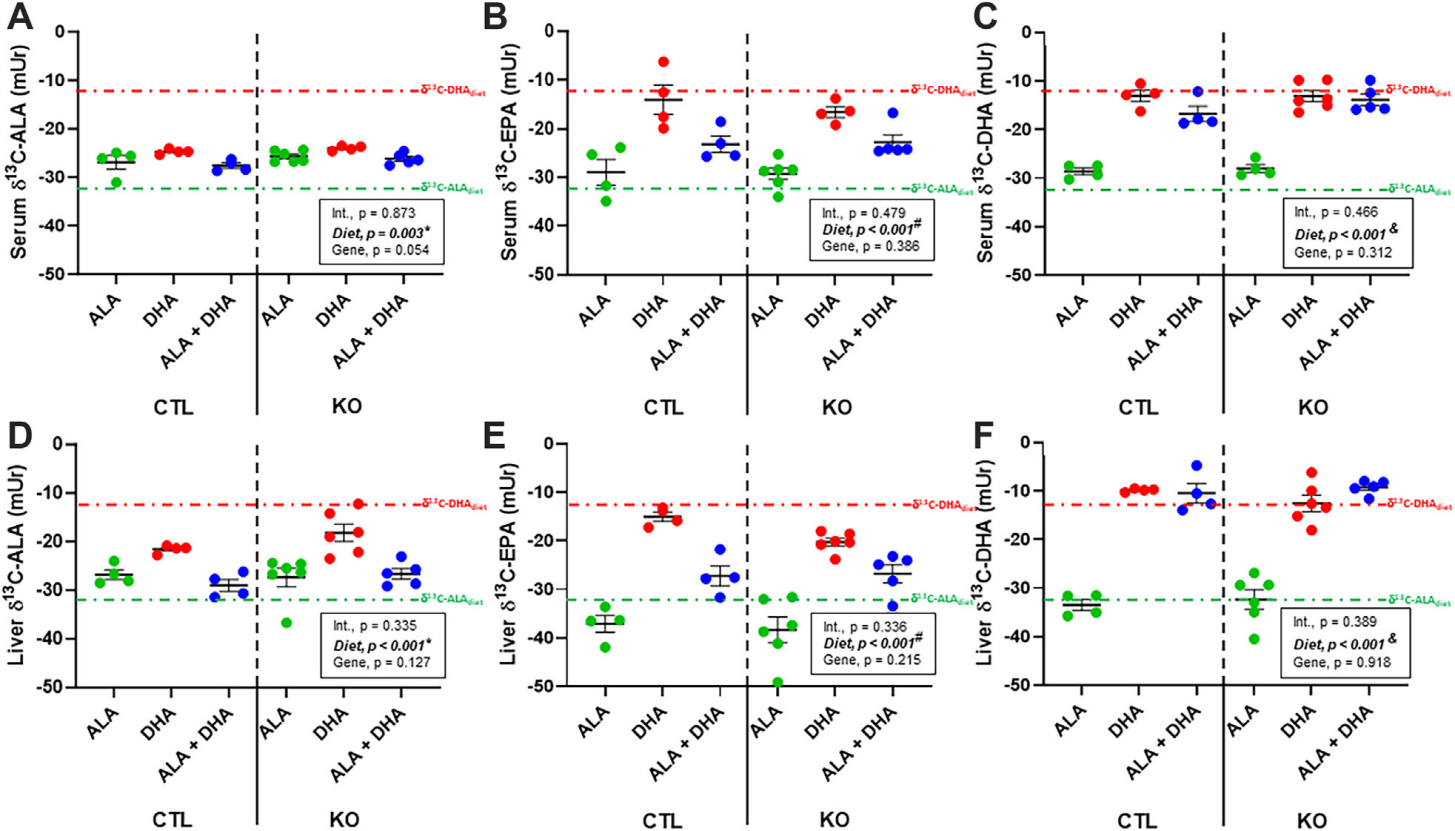
ALA, EPA, and DHA carbon-13 levels (δ^13^C) of male liver-specific *Elovl2* KO and control mice fed 4 weeks of: (1) ALA only (ALA), (2) DHA only (DHA) or (3) ALA + DHA diets following a 4-week ALA run-in diet. A: serum ALA, (B) serum EPA, (C) serum DHA, (D) liver ALA, (E) liver EPA and (F) liver DHA. Normality was determined using a Shapiro-Wilk test and nonnormally distributed data were log transformed prior to further statistical analysis. Significant interaction and main effects of genotype and diet were determined by two-way ANOVA. Main effects of diet were further assessed by Tukey’s post hoc test, where for serum and liver δ^13^C-ALA (∗) represents a diet effect of (ALA = ALA + DHA) < DHA, for serum and liver δ^13^C-EPA (^#^) represents a diet effect of ALA < ALA + DHA < DHA and for serum and liver δ^13^C-DHA (^&^) represents a diet effect of ALA < (DHA = ALA + DHA). All values are reported in means ± SEM (n = 4–6). δ^13^C, carbon-13 abundance; δ^13^C-ALA_diet_, mean δ^13^C (mUr) of ALA and ALA+DHA diets; δ^13^C-DHA_diet_, mean δ^13^C (mUr) of DHA and ALA+DHA diets; ALA, α-linolenic acid, 18:3n-3; DHA, docosahexaenoic acid, 22:6n-3; *Elovl2*, elongation of very long chain 2l EPA, eicosapentaenoic acid, 20:5n-3.

Serum (Fig. 6A–C) and liver (Fig. 6D–F) δ^13^C-ALA, EPA and DHA were determined for liver-specific *Elovl2* KO and control mice fed ALA, DHA, or ALA + DHA diets for 4 weeks following a 4-week ALA only diet. There were no effects of genotype on serum or liver δ^13^C-ALA, δ^13^C-EPA or δ^13^C-DHA (*P* > 0.05). Significant main effects of diet were determined for serum δ^13^C-ALA (*P* = 0.003), serum δ^13^C-EPA (*P* < 0.001), serum δ^13^C-DHA (*P* < 0.001), liver δ^13^C-ALA (*P* < 0.001), liver δ^13^C-EPA (*P* < 0.001), and liver δ^13^C-DHA (*P* < 0.001). In both the serum and liver, δ^13^C-ALA was higher in the DHA only group (−24.4 ± 0.2 and −19.5 ± 1.2 mUr ± SEM, respectively) compared to both the ALA group (−26.2 ± 0.2 and −27.1 ± 1.2 mUr, respectively) and the ALA + DHA group (−26.8 ± 0.4 and −27.7 ± 0.9 mUr, respectively), with the ALA and ALA + DHA groups not different (*P* > 0.05). In both the serum and liver, δ^13^C-EPA was highest in the DHA only diet (−15.3 ± 1.6 and −18.2 ± 1.0, respectively), followed by the ALA + DHA diet (−23.0 ± 1.0 and −27.0 ± 1.3, mUr, respectively) and the ALA only diet (−29.1 ± 1.2 and −37.8 ± 1.7 mUr, respectively). In both the serum and liver, δ^13^C-DHA was higher in the DHA only and ALA + DHA diets (−9.8 ± 0.9 to −15.1 ± 1.0 mUr) compared to the ALA only diet (−28.3 ± 0.5 and −32.8 ± 1.2 mUr, respectively). Compared to serum and liver δ^13^C-EPA, a similar response for δ^13^C-DPAn-3 was found, but with an additional small but significant genotype effect (supplemental Fig. S3).

### DHA inhibition of ELOVL2/5 (EPA → DPAn-3) by enzyme activity competition assay

**Fig. 7:**
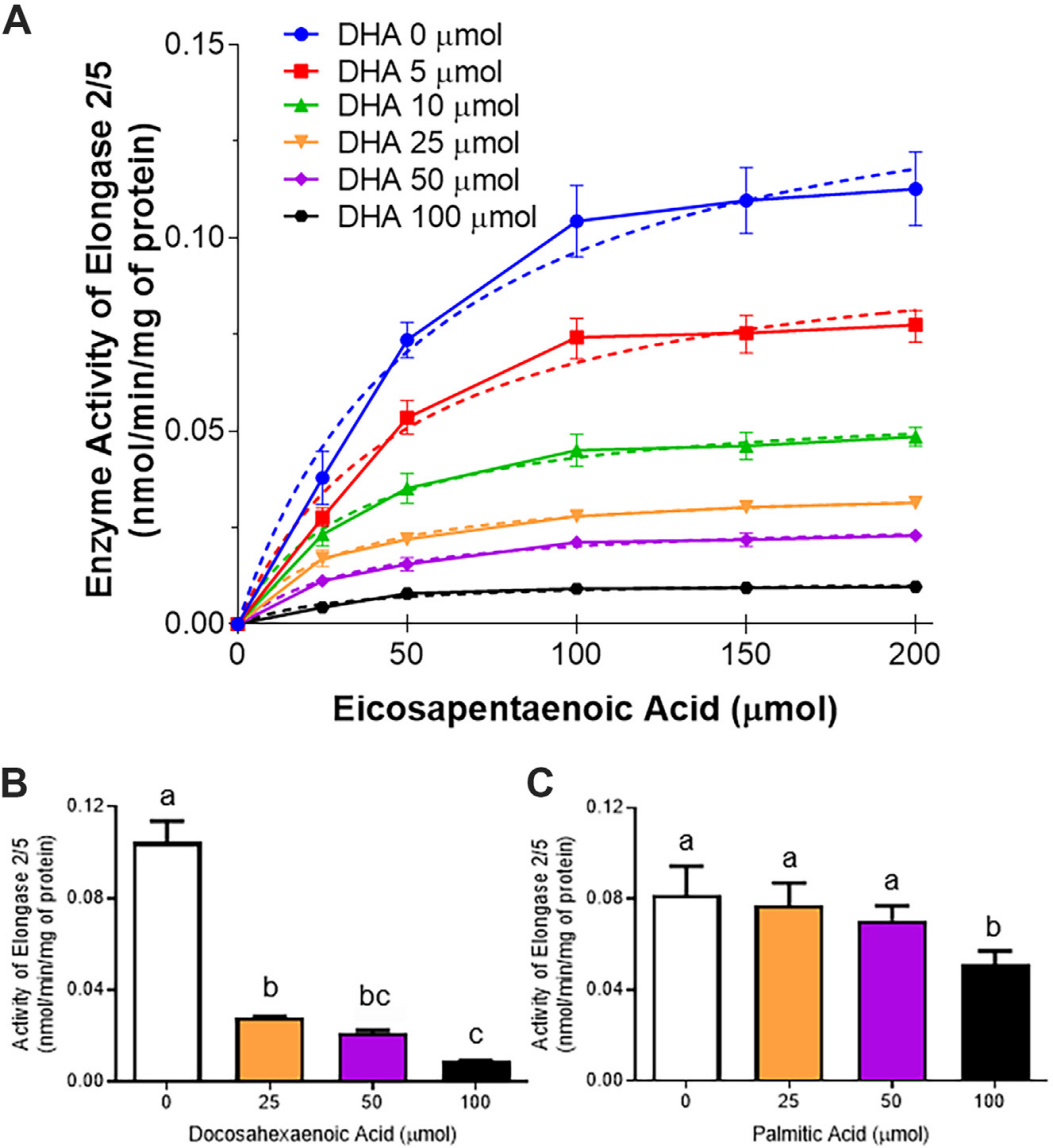
Enzyme activity assays for inhibition EPA elongation to DPAn-3 (ELOVL2/5) by DHA and palmitic acid. A: Enzyme competition assay in the presence of DHA (n = 3 per data point), and enzyme activity of elongase 2/5 in the presence of 0, 25, 50, and 100 μmol of either (B) DHA (n = 3), or (C) palmitic acid (control, n = 4–5) in isolated microsomes. Normality was determined using a Shapiro-Wilk test and nonnormally distributed data were log transformed prior to further statistical analysis. Different letters (a, b, and c) represent statistically significant differences (*P* < 0.05) between DHA or palmitic acid treatment levels as determined by one-way ANOVA followed by a Tukey’s HSD post hoc test. All values are reported in means ± SEM (n = 3–5). DHA, docosahexaenoic acid, 22:6n-3; DPAn-3, n-3 docosapentaenoic acid, 22:5n-3; EPA, eicosapentaenoic acid, 20:5n-3; HSD, honestly significant difference.

Using microsomal isolations from chow-fed BALB/c mice, a competition assay for the enzyme activity (nmol/min/mg protein ± SEM) of EPA to DPAn-3 elongation (ELOVL2/5) was assessed with DHA (0, 5, 10, 25, 50, and 100 μmol) as the inhibitor and fitted to a Michaelis-Menten model (Fig. 7A). Increasing DHA levels resulted in a decrease of V_max_ from control (0.152 ng/min/mg) to 0.102, 0.058, 0.036, 0.027, and 0.012 in the presence of 5, 10, 25, 50, and 100 μmol of DHA. The K_m_ or the amount of substrate required to reach ½ V_max_, were 57.6, 50.3, 33.5, 29.5, 35.8, and 31.3 μmol of EPA when in the presence of 0, 5, 10, 25, 50, and 100 μmol DHA, respectively. A separate set of liver samples were used to isolate microsomes and additional enzyme inhibition assays were performed in the presence of 0, 25, 50, and 100 μmol of DHA (Fig. 7B) or a palmitic acid control (Fig. 7C). ELOVL2/5 enzyme activity was significantly lower (*P* < 0.05) than control (0.104 ± 0.005 ng/min/mg) when exposed to 25 (0.028 ± 0.0003 ng/min/mg), 50 (0.021 ± 0.0007 ng/min/mg), and 100 μmol (0.009 ± 0.0001 ng/min/mg) of DHA, with 100 μmol of DHA also being significantly lower than 25 μmol DHA. Conversely, ELOVL2/5 enzyme activity in the presence of 25 (0.077 ± 0.004 ng/min/mg) and 50 μmol (0.071 ± 0.003 ng/min/mg) palmitic acid was not different (*P* > 0.05) than control (0.082 ± 0.006 ng/min/mg), but 100 μmol (0.051 ± 0.003 ng/min/mg) palmitic acid significantly decreased (*P* < 0.05) activity compared to all other levels of palmitic acid.

### Change in plasma EPA levels in humans supplemented with 3 g/day of DHA for 12 weeks

**Fig. 8:**
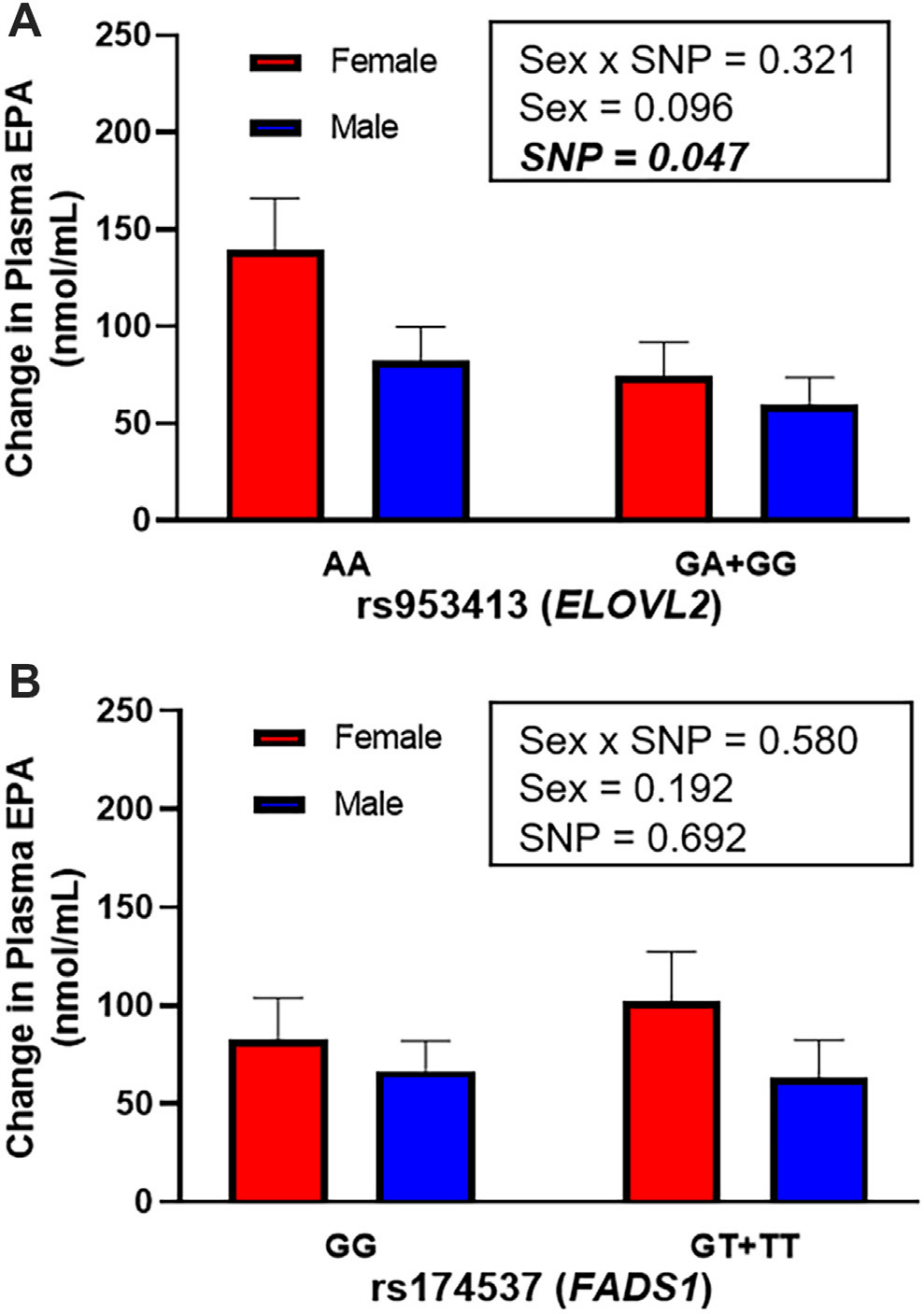
Change from baseline in plasma EPA concentrations following 12 weeks of 3 g/day DHA supplementation by sex and genotype. A: rs953413 and (B) rs174537. Normality was determined using a Shapiro-Wilk test and nonnormally distributed data were log transformed prior to further statistical analysis. Statistical significance (*P* < 0.05) for interaction or main effects of sex and genotype were determined by two-way ANOVA. Values expressed as means ± SEM. For rs953413, n = 4 (AA female), 4 (AA male), 11 (GA + GG female), and 11 (GA + GG female), and for rs174537, n = 8 (GG female), 8 (GG male), 7 (GT + TT female), and 7 (GG + TT male). DHA, docosahexaenoic acid, 22:6n-3; *ELOVL*, elongation of very long chain; EPA, eicosapentaenoic acid, 20:5n-3; *FADS*, fatty acid desaturase.

The role of sex and genetics on changes in plasma EPA levels of young women and men was assessed following 12 weeks of DHA supplementation (Fig. 8). An *ELOVL2* SNP, rs953413, showed no significant interaction effect (*P* = 0.321), nor a significant effect of sex (*P* = 0.096) (Fig. 8A). However, a significant effect (*P* < 0.05) of rs953413 was identified resulting in a 66% larger increase in plasma EPA levels for those individuals with the AA genotype (111 ± 18, nmol/ml ± SEM) compared to those with the GA or GG genotype (67 ± 11.1 nmol/ml). Finally, the interaction of sex with the *FADS1* SNP, rs174537, was also assessed and revealed no significant interaction effects (*P* = 0.580) or main effects of sex (*P* = 0.192) or rs174537 (*P* = 0.692) (Fig. 8B).

## Discussion

In this study, we used various models to determine the mechanism(s) by which DHA synthesis is downregulated by dietary DHA intake. These include (1) dietary studies demonstrating increases in EPA derived from both ALA and DHA in WT, and control and liver-specific *Elovl2* KO mice following DHA feeding, (2) gene expression, protein content, and enzyme activity of PUFA desaturase/elongase enzymes in WT mice following DHA feeding, (3) enzyme inhibition assays in microsomal isolations to assay the elongation of EPA to DPAn-3 in the presence of DHA, and (4) a human SNP in the *ELOVL2* gene in DHA supplemented adults that contributes to increased plasma EPA. This cumulation of evidence supports our previous animal (36) and human (24) studies demonstrating that the rise in EPA following dietary DHA is due to reduced downstream EPA metabolism and not increased retroconversion of DHA to EPA, and identifies elongation of EPA, in particular by ELOVL2, as a novel and important inhibitory target of DHA via a negative feedback pathway. Collectively, our work demonstrates for the first time that dietary DHA downregulates its own synthesis in the liver by inhibiting EPA elongation.

We demonstrated no significant effects of the ALA, DHA, and ALA + DHA diets on brain DHA concentrations, but with CSIA determined that in the absence of dietary DHA the brain receives DHA synthesized from ALA, confirming our previous results in C57Bl/6J and BALB/c mice (37, 46, 47). Furthermore, the pattern for brain δ^13^C-DHA (DHA > ALA+DHA > ALA, Fig. 1) matches serum (Fig. 6C) and, although brain δ^13^C-DHA is overall lower due to a relatively slow DHA turnover rate, given enough time we expect brain δ^13^C-DHA to represent the δ^13^C-DHA supplied from serum, as previously shown (47). We then recapitulated results from prior studies in animals (25, 26, 27, 28, 29, 30, 31, 32) and humans (16, 17, 18, 19, 20, 21, 22, 23, 24) demonstrating increased plasma/serum and liver EPA following DHA feeding (Fig. 2). However, EPA levels only increased with DHA feeding in the presence of ALA and were not different between ALA or DHA only fed animals, demonstrating that ALA is necessary to increase EPA with DHA feeding. Similarly, rats fed a 2.25% ALA (in total fat) diet or a 2% DHA diet did not differ in whole body EPA concentrations, while EPA levels in tissues of rats provided both diets were higher when compared to those provided a low ALA diet (0.25%) (48). However, serum/liver DPAn-3 in the DHA group was lower than either ALA group, possibly due to the presence of only one dietary source of DPAn-3 in the DHA group combined with inhibition of EPA elongation. Conversely, there is still only one DPAn-3 source in the ALA group, but the lack of inhibition allows DPAn-3 to be synthesized freely from EPA resulting in higher DPAn-3 in the ALA compared to DHA group.

The necessity of combining ALA + DHA to increase EPA provides critical information to the mechanism of EPA’s increase. Since ALA is a precursor to EPA, any increase in EPA must then be the result of slowed metabolism of ALA-derived EPA. Furthermore, we utilized high δ^13^C-DHA of −11.0 to −14.0 mUr compared to the flaxseed-derived δ^13^C-ALA of −31.2 to −34.0 mUr. This allows for identifying the source underlying EPA accumulation, either from ALA or DHA, in each of the diets. As such, liver and serum δ^13^C-EPA from BALB/c mice were not different between the ALA and ALA + DHA diets indicating most of the increased EPA came from ALA (Fig. 3). These results were predicted by our previous rat (36) and human (24) studies. However, although our C57Bl/6J control mice from the *Elovl2* KO study show the same pattern for fatty acid levels (Fig. 5), serum, and liver δ^13^C-EPA levels suggest a mixed contribution of ALA and DHA (Fig. 6), discussed in more detail later. In the BALB/c mice, not until ALA is removed from the diet does δ^13^C-EPA increasess toward that of DHA, revealing that retroconversion rates are slow and DHA is not a major source of EPA until ALA intakes are low.

Although no known pathway exists to convert DHA to ALA, the serum and liver δ^13^C-ALA was higher in the DHA group suggesting the possibility of a pathway when ALA intakes are very low. Alternatively, β-oxidation prefers ^12^C molecules over ^13^C molecules, a process known as carbon isotopic fractionation (49), which could explain the higher δ^13^C-ALA remaining in the body. Furthermore, although statistical analyses were not performed to compare serum and liver δ^13^C values, there may be a trend for lower δ^13^C-n-3 PUFA compared to the liver, suggesting that adipose, with a DHA half-life of 5–8 days or up to 30 days from dietary DHA or ALA, respectively (37), may still be contributing to serum δ^13^C levels after 28 days of high δ^13^C-DHA feeding. Although the liver δ^13^C-ALA (but not serum δ^13^C-ALA) was unexpectedly higher in the ALA compared to ALA + DHA group, this did reflect the δ^13^C-ALA differences in the diets and could again be indicative of an impact of stored adipose n-3 PUFA that differentially affects serum and liver δ^13^C values.

Both DHA diets reduced mRNA expression, protein content, and enzyme activity of FADS2, but only affected protein content and enzyme activity of FADS1 (Fig. 4). This suggests that although increases in DHA appear to affect the activity of both desaturase enzymes, the mechanism by which this occurs may be transcriptional for FADS2 and translational for FADS1. However, with the putative rate-limiting FADS2 reaction and the only FADS1 reaction occurring prior to EPA in the pathway, neither of these enzymes are expected to be involved in EPA accumulation with DHA feeding. Alternative to the desaturases (FADS1/2), the elongases (ELOVL2/5) contribute relatively equally to the elongation of EPA to DPAn-3 and have been identified as critical control points in the DHA biosynthesis pathway (50). DHA compared to ALA feeding appeared to lower mRNA expression of *Elovl2* and *Elovl5*; however, significance was not reached in the ALA + DHA group, and this lowering did not translate to lower protein content. Furthermore, DHA feeding did not affect ELOVL5 activity or the combined ELOVL2/5 activity; however, ELOVL2 activity was lower in both DHA fed groups compared to the ALA fed group. There are limitations of the enzyme activity assay: (1) microsomes are fragmented pieces of the endoplasmic reticulum that may not represent the normal structural support for the elongase and desaturase enzymes, and (2) the fatty acid composition that the isolated microsomes are exposed to may be altered compared to the intact cell. Nevertheless, we demonstrated that ELOVL2 activity is lowered independent of changes in protein content, suggesting that DHA exerts posttranslational modifications on ELOVL2, but not ELOVL5.

With the enzyme activity assay indicating ELOVL2 inhibition with increasing DHA levels, we repeated the ALA, DHA, or ALA + DHA feeding study in liver-specific *Elovl2* KO and control mice to investigate how n-3 PUFA levels and δ^13^C compare in the presence or absence of a functional liver Elovl2. Liver is the primary active tissue of the n-3 PUFA pathway and the most abundant tissue for expression of *Elovl2* in rodents (51). Despite being maintained on different diets, a whole body *Elovl2* KO model yielded similar serum and liver EPA and DHA patterns compared to our liver-specific *Elovl2* KO (52), suggesting that a whole body *Elovl2* KO is metabolically similar to knocking out the liver only. In both liver and serum of our liver-specific KOs, EPA and DPAn-3 levels were higher in the ALA-fed KO mice compared to control, indicating the expected accumulation of ELOVL2 substrates as a result of liver *Elovl2* KO. *Elovl2* KO did not result in any additional increase in liver EPA levels following DHA or ALA + DHA feeding compared to control mice. The serum results are nearly identical to the liver, with one exception, the ALA + DHA fed KO mice displayed significantly higher serum EPA levels compared to control mice on the same diet. Furthermore, the serum δ^13^C-DHA pattern matches that of the brain nearly identically. Importantly, there were no interaction (diet × genotype) or genotype effects for serum or liver δ^13^C-EPA revealing that the source of the increase in EPA of DHA-fed mice was the same in both KO and control groups. This indicates that the mechanism of the increase in EPA, KO/inhibition of elongation of EPA, may also the same. Incredibly, our results indicate that DHA feeding may have a similar effect on liver ELOVL2 activity as compared with complete liver *Elovl2* KO. However, the diet effect achieved in the C57Bl/6J mice shows intermediate δ^13^C-EPA levels in the ALA + DHA group, while the two DHA fed groups in the BALB/c mice are not different suggesting contribution from both ALA and DHA to the increased EPA. When there are two sources of fatty acids, such as in our ALA + DHA diet group, the relative contribution of ALA and DHA to EPA can be calculated (34, 36). As a result, we estimate that 62%–95% of EPA was derived from serum ALA in BALB/c mice (supplemental Fig. S4A), while 63%–90% of EPA was derived from serum ALA in the C57Bl/6J mice regardless of genotype (supplemental Fig. S4B). Supportive of these findings, we previously applied the same modeling to estimate the contribution of dietary ALA to liver EPA to be 63% in Long-Evans rats on an ALA + DHA diet (36), indicating that the majority of the increase in EPA with DHA feeding is derived from ALA. Furthermore, the similarities between control and KO mice indicate that with DHA feeding less ALA is being converted past EPA and into DHA, which when combined with the brain δ^13^C-DHA values indicate that increased DHA shuts down its own synthesis in the liver to prevent redundancy of two brain DHA supplies.

Although our feeding studies in WT, control, and *Elovl2* KO mice clearly show that EPA elongation is inhibited by DHA, the nature of this inhibition cannot be elucidated by fatty acid concentrations and δ^13^C values alone. Therefore, we isolated microsomes from chow-fed mice to perform a competition assay and identify how DHA (0–100 μmol) is inhibiting EPA (0–200 μmol) elongation to DPAn-3. The V_max_ of the EPA elongation reaction decreased with each successive dosage increase in DHA, demonstrating that this is not a competitive inhibition, and DHA does not occupy the same enzymatic site as EPA. This is contrary to what we might expect to see with known ELOVL2 substrates such as DPAn-3, arachidonic acid (20:4n-6), or adrenic acid (22:4n-6) (53). The K_m_, which represents ½ of the V_max_, decreased from 0 to 10 μmol DHA (slope = −0.2408, *r*^2^ = 0.9505) and then plateaued from 10 to 100 mmol DHA (slope = −0.0058, *r*^2^ = 0.0069) (Fig. 7A). Combined with decreasing V_max_ (1) a decreasing K_m_ reveals uncompetitive inhibition where the inhibitor binds to the enzyme after the enzyme-substrate complex has been formed to inhibit the reaction from occurring, and (2) a constant K_m_ indicates noncompetitive inhibition where the inhibitor binds to the enzyme preventing the substrate from binding (54). Therefore, at low DHA levels, DHA binds to the ELOVL2 enzyme after EPA has bound to form the enzyme-substrate complex and alters ELOVL2 activity by uncompetitive inhibition. At higher DHA levels, DHA either (1) binds to the elongase prior to binding EPA, preventing EPA from binding to the active site on ELOVL2, or (2) binds to ELOVL5 adding additional, noncompetitive, inhibition of EPA elongation. Due to limitations in the enzyme affinity for EPA in this assay, supraphysiological EPA levels must be used to obtain measurable DPAn-3 product levels. Nevertheless, the relative proportions of the DHA inhibitor to EPA substrate levels in the assay (from 1:40 up to 4:1 DHA:EPA) are comparable to previously reported physiological liver DHA:EPA proportions (55, 56), although still lower than shown in our mouse livers (minimum 12:1 in the control mice).

To test our animal findings in humans, we also performed a secondary analysis of a previously published randomized controlled trial (RCT) in young men and women supplemented 3 g/day DHA for 12 weeks (24, 40). In that study, DHA supplementation increased plasma EPA levels from 59 to 137 nmol/ml, however, the δ^13^C-EPA values did not change despite the significantly higher δ^13^C-DHA of the supplement. If increased retroconversion had occurred, the δ^13^C-EPA in plasma would have shifted toward that of δ^13^C-DHA in the supplement; however, the lack of change in plasma δ^13^C-EPA indicated that the source of the higher EPA remained primarily ALA. In the current study, we stratified the data by sex and the *ELOVL2* SNP rs953413 and found that the change in plasma EPA was potentially dependant on rs953413, with the AA genotype resulting in a significantly higher EPA increase compared to the combined GA + GG genotype. Although a sex effect trend (*P* = 0.096) was revealed that may be driving these findings, due to the secondary nature of our analysis we may be underpowered to detect this effect, and additional appropriately powered clinical trials are warranted. However, the role of this rs953413 SNP, in addition to rs3734398 and rs2236212 *ELOVL2* SNPs, has been assessed in a mixed sex population following 6 months of 0, 0.45, 0.9, and 1.8 g/d fish oil supplementation (57). Following the 1.8 g/d dose only, there were significant genotype effects for EPA levels for each of the aforementioned SNPs, with the GA + AA genotype of rs953413 displaying significantly higher EPA compared to the GG genotype. However, with mixed EPA + DHA fish oil supplementation, firm conclusions on the source of the increased EPA are not possible. The rs953413 SNP is located within an evolutionarily conserved enhancer region, and acts as an important regulator for *ELOVL2* expression, particularly via the cooperation of FOXA1/FOXA2 and HNF4αto increase *ELOVL2* expression in the G allele (58). If DHA supplementation was to decrease FOXA1, FOXA2, and/or HNF4α, this could result in downregulation of *ELOVL2* expression in the GG genotype, thereby resulting in a relatively larger increase in EPA for those with the AA genotype. However, to our knowledge, the effects of DHA supplementation on these protein levels have not been investigated and represents an intriguing avenue of research.

The inhibition of EPA metabolism with DHA supplementation has significant implications for our understanding of how tissues, like the brain, obtain DHA. Our results demonstrate that when DHA is consumed in the diet, it inhibits its own synthesis from ALA by decreasing the elongation of EPA which then accumulates. Thus, when liver cannot be assessed directly, such as in clinical studies, augmentation of EPA coupled with CSIA can be used as a surrogate to measure the inhibition of EPA elongation. Conversely, when DHA is absent in the diet, as would be common in vegans and those who do not consume fish, EPA elongation is not inhibited, and ALA can be used to synthesize DHA. Future work examining the effects of development, genetics, and stress on the responsiveness of this pathway are warranted. Beyond this, a literature is emerging suggesting that DHA may alter the effects from EPA (59, 60). RCTs using mixed EPA/DHA supplements to reduce cardiovascular disease end points have been unsuccessful (61, 62) while two RCTs supplementing EPA alone reported cardiovascular benefits (63, 64). Similar meta-analysis findings have been identified for major depression (65). Our mechanistic findings could help explain the contradictory RCT findings. EPA, when supplemented alone, is free for downstream conversion to DPAn-3 and its bioactive metabolites. However, when combined with DHA, EPA metabolism is inhibited and less available for potentially antiinflammatory metabolite production.

In summary, we have shown clearly that EPA accumulation with increasing DHA levels results predominantly from impaired EPA elongation to DPAn-3 in a negative feedback system via the uncompetitive inhibition of the enzymes involved, likely liver ELOVL2. Furthermore, individuals with the AA genotype in the *ELOVL2* SNP, rs953413, show larger increases in EPA with DHA supplementation. Although we did not reveal any sex effects, a trend was observed, and we may have been underpowered to detect these differences, and future studies should focus on investigating the sex-specific effects of dietary DHA inhibition of EPA elongation. In conclusion, the inhibition of liver DHA synthesis by dietary DHA reveals a mechanism by which dietary DHA controls the source and delivery of DHA to the brain.

## Data availability

Additional data is to be shared upon request from the corresponding author (A. H. M.).

## Supplemental data

This article contains supplemental data.

## Conflict of interest

D. M. M. has received research grants from the Canola Council of Canada and the Dairy Farmers of Canada. R. P. B. has received industrial grants, including those matched by the Canadian government, and/or travel support related to work on brain fatty acid uptake from Arctic Nutrition, Bunge Ltd, DSM, Fonterra, Mead Johnson, and Nestle, Inc. Moreover, R. P. B. is on the executive of the International Society for the Study of Fatty Acids and Lipids and held a meeting on behalf of Fatty Acids and Cell Signaling, both of which rely on corporate sponsorship. R. P. B. has given expert testimony in relation to supplements and the brain and holds the Canada Research Chair in Brain Lipid Metabolism. A. H. M. is a board member of the ISSFAL. None of the other authors report a conflict of interest related to research presented in this article.
