# Supplementary material for: Dietary docosahexaenoic acid (DHA) downregulates liver DHA synthesis by inhibiting eicosapentaenoic acid elongation
Source: J Lipid Res. 2024 Apr 20;65(6):100548. doi: 10.1016/j.jlr.2024.100548 (PMC11126934; doi:10.1016/j.jlr.2024.100548)
Supplement: Supplemental data [file mmc1.docx]

**Supplemental Table 1** – Fatty Acid Composition and δ^13^C of Mouse Diets

|  | Diet Compositions | | |
| --- | --- | --- | --- |
| Fatty Acid | ALA | DHA | ALA + DHA |
| 12:0 | 28.9 ± 0.3 | 29.5 ± 0.2 | 29.7 ± 0.1 |
| 14:0 | 12.4 ± 0.1 | 12.6 ± 0.1 | 12.7 ± 0.1 |
| 16:0 | 9.13 ± 0.06 | 9.16 ± 0.07 | 9.19 ± 0.06 |
| 18:0 | 8.49 ± 0.06 | 8.50 ± 0.05 | 8.51 ± 0.03 |
| 18:1n-7 | 0.30 ± 0.004 | 0.29 ± 0.003 | 0.29 ± 0.003 |
| 18:1n-9 | 7.95 ± 0.05 | 7.78 ± 0.05 | 5.7 ± 0.01 |
| 18:2n-6 | 28.2 ± 0.18 | 27.6 ± 0.18 | 27.4 ± 0.06 |
| 18:3n-3 | 2.20 ± 0.02 | 0.15 ± 0 | 2.15 ± 0.01 |
| *δ^13^C-18:3n-3* | *-31.2 ± 0.4* | *n.d* | *-34.0 ± 0.8* |
| 22:6n-3 | n.d. | 1.85 ± 0.01 | 1.92 ± 0.02 |
| *δ^13^C-22:6n-3* | *n.d.* | *-14.0 ± 1.3* | *-11.0 ± 0.3* |
|  |  |  |  |
| **ALL SFA** | 61.0 ± 0.2 | 61.8 ± 0.2 | 62.1 ± 0.1 |
| **ALL MUFA** | 8.45 ± 0.06 | 8.29 ± 0.05 | 6.21 ± 0.02 |
| **N-6 PUFA** | 28.2 ± 0.2 | 27.6 ± 0.2 | 27.4 ± 0.1 |
| **N-3 PUFA** | 2.2 ± 0.02 | 2.00 ± 0.01 | 4.07 ± 0.02 |
| **ALL PUFA** | 30.4 ± 0.2 | 29.6 ± 0.2 | 31.5 ± 0.1 |

Fatty acid compositions reported as weight percent and δ^13^C as milliUrey (mUr). All values are reported as means ± SEM (n = 3), with fatty acid levels reported as % of fatty acid in total fatty acids and carbon-13 content (δ^13^C) reported in milliUrey (mUr). ALA – α-linolenic acid, 18:3n-3; DHA – docosahexaenoic acid, 22:6n-3; MUFA – monounsaturated fatty acid; n.d. – not detected; PUFA – polyunsaturated fatty acids; SFA – saturated fatty acids.

**Suppl. Figure 1**

**
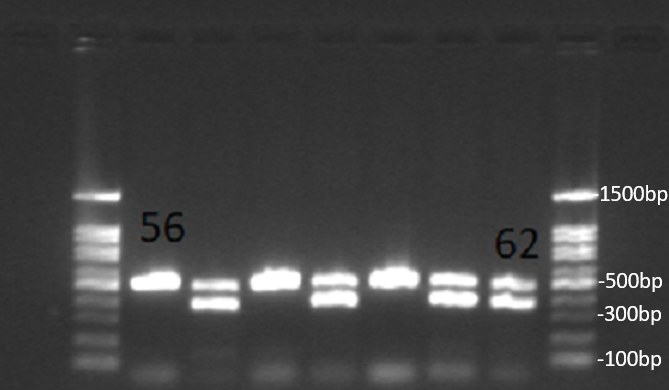
**

**Supplemental Figure 1** – Representative agarose gel for identification of Cre+ mice (386 bp) in mice with codes 57, 59, 61 and 62 and β-actin+ mice (positive control, 502 bp) in all mice with reference to a DNA ladder including 100 – 1500 bp bands.

**Suppl. Figure 2**

**A**

**
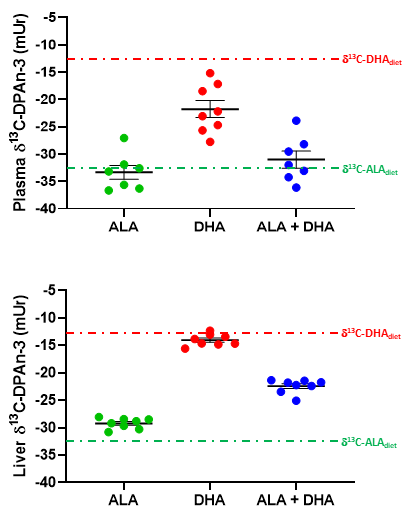
**

a

b

c

a

b

a

**B**

**Supplemental Figure 2** – Carbon-13 levels (δ^13^C) for A) plasma DPAn-3 and B) liver DPAn-3 of male BALB/c mice fed 4 weeks of: 1) ALA only (ALA), 2) DHA only (DHA) or 3) ALA + DHA diets following a 4-week ALA run-in diet. Normality was determined by Shapiro-Wilk test for normality and non-normally distributed data was log transformed prior to further statistical analysis. Different letters represent statistically significant differences (p < 0.05) between diets and were determined by one-way ANOVA followed by a Tukey’s HSD post hoc test. All values are reported in means ± SEM (n = 7 – 8). ALA – α-linolenic acid, 18:3n-3; δ^13^C-ALA_diet_ – mean δ^13^C (mUr) of ALA and ALA+DHA diets; δ^13^C-DHA_diet_ – mean δ^13^C (mUr) of DHA and ALA+DHA diets; DHA – docosahexaenoic acid, 22:6n-3; DPAn-3 – n-3 docosapentaenoic acid, 22:5n-3.

**Suppl. Figure 3**

**
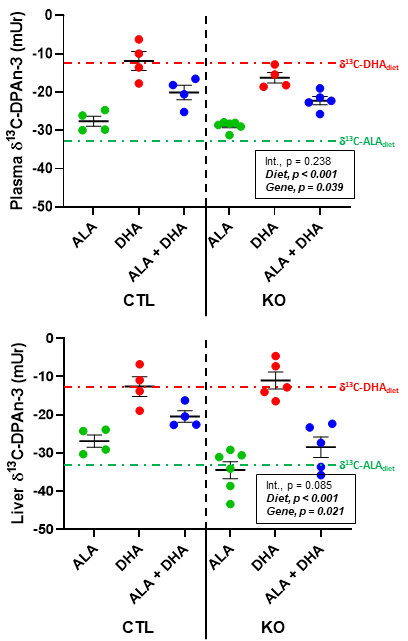
**

**Supplemental Figure 3** – Carbon-13 levels (δ^13^C) for A) plasma DPAn-3 and B) liver DPAn-3 of male liver-specific *Elovl2* knockout and control mice fed 4 weeks of: 1) ALA only (ALA), 2) DHA only (DHA) or 3) ALA + DHA diets following a 4-week ALA run-in diet. Normality was determined using a Shapiro-Wilk test and non-normally distributed data was log transformed prior to further statistical analysis. Significant interaction and main effects of genotype and diet were determined by two-way ANOVA. Main effects of diet were further assessed by Tukey’s Post-Hoc test. All values are reported in means ± SEM (n = 4 – 6). ALA – α-linolenic acid, 18:3n-3; δ^13^C-ALA_diet_ – mean δ^13^C (mUr) of ALA and ALA+DHA diets; δ^13^C-DHA_diet_ – mean δ^13^C (mUr) of DHA and ALA+DHA diets; DHA – docosahexaenoic acid, 22:6n-3; DPAn-3 – n-3 docosapentaenoic acid, 22:5n-3; *Elovl2* – elongation of very-long chain 2.

**Suppl. Figure 4**

**A**

**
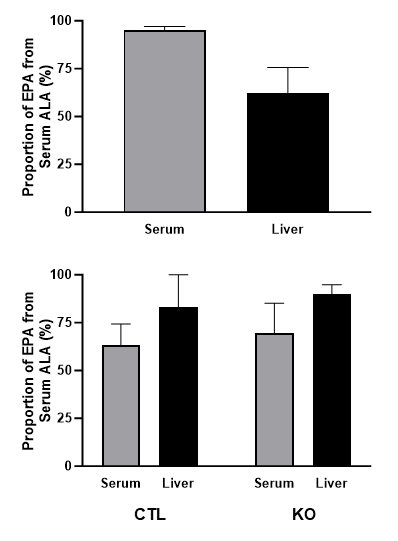
**

**B**

**Supplemental Figure 4** – Proportion of serum and liver EPA derived from serum ALA in A) male BALB/c mice and B) male liver-specific *Elovl2* knockout and control mice fed 4 weeks of ALA + DHA following a 4-week ALA run-in diet. All values are reported as means ± SEM (n = 4 – 8). ALA – α-linolenic acid, 18:3n-3; DHA – docosahexaenoic acid, 22:6n-3; EPA – eicosapentaenoic acid, 20:5n-3; *Elovl2* – elongation of very-long chain 2.
